# CPT-II deficiency needs to be detected in army personnel

**DOI:** 10.1016/j.ymgmr.2018.05.005

**Published:** 2018-05-30

**Authors:** Josef Finsterer

**Affiliations:** Krankenanstalt Rudolfstiftung, Vienna, Austria

**Keywords:** Mitochondrial, SANDO, POLG1, Sudden cardiac death, Takotsubo

We read with interest the case report by Balasubramanian et al. about a 35 yo female with CPT-II deficiency with a positive family history for the disease, who served in the army for 12 y despite typical clinical manifestations of the disorder [1]. We have the following comments and concerns.

The most interesting point of this case is that the patient could join the army despite her conspicuous history. She had exercise intolerance since age 12 y but could begin with military service at age 18 y. She served in the army during 12 y although she experienced frequent episodes of muscle weakness, myalgias, and myoglobinurea. Did the patient avoid reporting the symptoms at the medical military entry investigation? Batteries of investigations prior to entering military service seem to be insufficient to detect patients with periodic metabolic muscle disease. If going undetected, such patients are exposed to an unnecessary risk for the community and the proband as well. Excessive training as in the military with a neuromuscular disorder may worsen clinical manifestations and may increase the speed of progression in such disorders [2]. The patient may be exposed to the unnecessary risk of acute kidney failure and she may take drugs, which may trigger further episodes of rhabdomyolysis. Additionally, she may undergo general anesthesia, which could trigger episodes of muscle weakness as well [3]. A further disadvantage of not knowing the correct diagnosis is the possibility of becoming pregnant and transmitting the disease without preconceptual reconnoitering. This is of particular importance in case of CPT-II since fetal CPT-II may be lethal due to sudden cardiac death [4]. CPT-II mutations may even compromise intrauterine development [5].

In summary, we recommend that army personnel is generally screened for creatine-kinase values after excessive training. In case of excessively high values, these patients should undergo further neurological work-up and should not serve in the military.

## Conflict of interest

There are no conflicts of interest.

## Funding

No funding was received.

## Author contribution

JF: design, literature search, discussion, first draft, SZ-M: literature search, discussion, critical comments.

## Ethical approval

The research has been given ethical approval.
